# An Ultrasound-Guided Thoracolumbar Erector Spinae Plane Block: An Experimental Preliminary Study in Horses

**DOI:** 10.3390/ani15152264

**Published:** 2025-08-01

**Authors:** Francisco Medina-Bautista, Irene Nocera, Antonia Sánchez de Medina, Chiara Di Franco, Angela Briganti, Juan Morgaz, María del Mar Granados

**Affiliations:** 1 Animal Medicine and Surgery Department, Universidad de Córdoba, 14014 Córdoba, Spain; tsmedina189@hotmail.com (A.S.d.M.); v92moroj@uco.es (J.M.); pv2grmam@uco.es (M.d.M.G.); 2 Department of Veterinary Sciences, University of Pisa, 56124 Pisa, Italy; chiara.difranco@phd.unipi.it (C.D.F.); angela.briganti@unipi.it (A.B.); 3 Institute of Life Sciences, Sant’Anna School of Advanced Studies, Via Santa Cecilia 3, 56127 Pisa, Italy; irene.nocera@santannapisa.it

**Keywords:** ultrasound, erector spinae plane block, horse, back

## Abstract

The objective of this study was to evaluate the feasibility and efficacy of the ultrasound-guided thoracolumbar erector spinae plane (TL-ESP) block in standing horses. A total of 24 injections (*n* = 12) were performed at the L1 level using either 0.1 mL/kg of saline solution (SS group) or 2% lidocaine (LID group). The block feasibility was assessed based on needle visualization and injection time, while efficacy was evaluated through craniocaudal and dorsoventral (DV) spread using the pinprick technique over 270 min. Desensitization was observed at least once in 100% of horses in the LID group and in 75% in the SS group (*p* = 0.001). However, in the SS group, desensitization was primarily limited to the Th18 metamer at the 2 cm DV position, with a shorter duration compared to the LID group. The block onset occurred at 22.5 (11.25–60) min in the LID group and at 5 (5–30) min in the SS group (*p* = 0.069). The number of affected metamers was significantly higher in the LID group (2 [1–3]) compared to the SS group (1 [1–2.25], *p* = 0.014). At the 2 cm DV point, the end of the block effect occurred at 135 (120–210) min in the LID group and at 60 (3.75–60) min in the SS group (*p* = 0.001). Needle visualization was excellent in 95.8% of cases, and the mean injection time was 2.5 (2–3) min. These findings confirm that the TL-ESP block is a feasible technique in standing horses. However, its effect is predominantly localized to dorsal dermatomes with a limited ventral spread. Future studies evaluating larger volumes and multiple injection sites are warranted to enhance its clinical applicability.

## Simple Summary

Impinging dorsal spinous processes and joint osteoarthrosis articular processes of the thoracolumbar vertebrae are a common cause of poor performance in horses, as the thoracolumbar junction is the most predisposed to impairments. A locoregional technique called the erector spinae plane block has been recently described in horses. This technique is an interfascial block where the anesthetic agent is injected between the fascia covering the erector spinae muscular complex and the transverse process of the vertebra. The injected volume can spread over at least five metamers when 0.3 mL/kg is injected into this interfascial plane, affecting both the dorsal and ventral nerve rami, as well as the epidural space, in a cadaveric model. Our study proved that this technique is also feasible in standing horses. Despite the fact that both epidural and ventral rami can be affected by this technique, it may be considered safe when using volumes of 0.1 mL/kg. The block, performed with 4 mg/kg of lidocaine in a total volume of 0.1 mL/kg, effectively desensitized the dorsal dermatomes, with a mean spread of two metamers. This study represents a step forward in supporting the potential clinical application of the erector spinae plane block in horses.

## 1. Introduction

Impinging dorsal spinous processes (DSPs) and osteoarthritis of the articular process joints are frequently diagnosed bone-related causes of back pain in horses [1,2,3,4]. Despite a growing body of evidence, there is still considerable controversy regarding the best therapeutic approach to DSPs in horses. A recent international survey reported a wide variability in treatment preferences among equine orthopedic specialists, with 32% of respondents using ultrasonographic guidance for local injections in the interspinous space—highlighting the increasing interest in ultrasound-guided approaches as a potentially safer and more accurate alternative to blind techniques [5]. In recent years, fascial plane blocks guided by ultrasound—where the local anesthetic is deposited within specific fascial layers to affect nerves traveling through them—have become increasingly popular [6,7,8,9,10]. In the past decade, the erector spinae plane (ESP) block has emerged as one of the most promising interfascial techniques for addressing thoracolumbar pain. The ESP block involves the injection of a local anesthetic into the fascial plane between the erector spinae muscles (iliocostalis, longissimus, and spinalis) and the transverse processes of the vertebrae, with the aim of desensitizing the dorsal spinal nerves [11]. Although this technique is well developed in humans, dogs, cats, and ruminants [11,12,13,14,15,16,17,18,19,20,21,22,23], evidence supporting its use in Equids remains limited. To date, only four publications have described the application of the ESP block in horses: three case reports—one performed under general anesthesia [24] and two in standing procedures [25,26]—and one cadaveric study [27]. The case reports describe successful analgesia with low injection volumes and no observable motor deficits; however, their anecdotal nature limits their generalizability. Despite being encouraging, case reports represent a low level of evidence (LoE 5) and typically involve a small number of animals under specific clinical conditions [28].

On the other hand, Delgado et al. [27] conducted a cadaveric study targeting the thoracolumbar region (T15) using 0.3 mL/kg of a solution consisting of a 50:1 ratio of 2% lidocaine and a yellow tissue dye, per injection. Despite achieving a high percentage of dorsal nerve staining (100%), they reported a 20% incidence of epidural spread—raising concerns about the safety of this block in standing procedures when local anesthetics are used. The same study also reported 21% ventral rami staining, suggesting potential for ventral analgesia, as has been described in humans [29,30]. Nonetheless, anatomical variations along the equine spine, the cadaveric nature of the study, and the discrepancy in volumes between that study and clinical reports limit the extrapolation of these results to live horses.

The primary aim of the present study is to describe the feasibility of performing an ESP block at the thoracolumbar level (TL-ESP) in standing, awake horses. The secondary objective is to assess its potential efficacy in producing dorsal desensitization and to evaluate whether the local anesthetic spreads ventrally along the flank. We hypothesize that the TL-ESP block is a feasible technique in standing horses and that it will desensitize the dorsal region without significant ventral diffusion or motor impairment.

## 2. Materials and Methods

### 2.1. Study Design

A prospective, randomized, double-blinded, and descriptive experimental trial was conducted. Horses were randomly assigned to receive either lidocaine (LID group) or saline solution (SS group) via the TL-ESP block.

Following approval from the local Animal Welfare and Ethics Committee (CEBAHCV 10/2023), twelve adult horses (mean ± SD: 456 ± 70 kg; age: 16 ± 7 years; body condition score: 4 [range 4–5] out of 9) were included in the study [31]. Of these, nine were mares (9/12; 75%) and three were stallions (3/12; 25%). Regarding temperament, nine horses (9/12; 75%) were classified as calm and well-handled, two (2/12; 16.7%) as restless and anxious, and one (1/12; 8.3%) as aggressive. All animals belonged to the Veterinary Teaching Hospital of the University of Córdoba.

Inclusion criteria comprised adult horses in good general health and body condition, with no previous history of back pain or trauma. The temperament of each horse was assessed prior to the beginning of the study [32]. Data from animals in which anatomical landmarks could not be clearly identified during ultrasound-guided (UG) scanning or hydrodissection were excluded from analysis.

### 2.2. TL-ESP Injection

**Figure 1:**
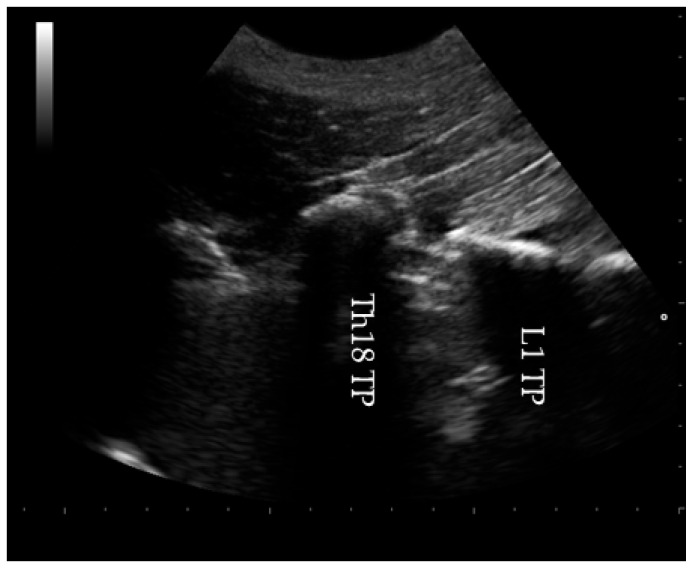
A parasagittal ultrasound image at the level of the eighteenth thoracic (Th18) and first lumbar (L1) transverse processes (TPs).

An area between the sixteenth thoracic (Th16) and the fifth lumbar (L5) vertebrae was clipped and aseptically prepared. A linear 5 MHz convex transducer (Esaote Mylab25, Genoa, Italy) was placed longitudinally over the spine, 5 cm parasagittal to the spine [25], to identify the injection site based on the difference in width between the transverse processes of the eighteenth thoracic (Th18) and the first lumbar (L1) vertebrae (Figure 1). The probe was then slightly shifted caudally to center the L1 transverse process within the acoustic window.

**Figure 2:**
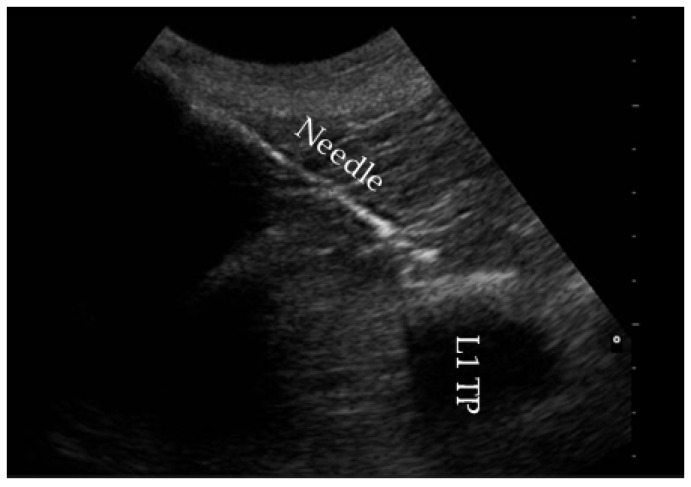
The craniocaudal direction of the needle towards the proximity of the first lumbar transverse process (L1 TP).

Once the injection site was identified, the skin and subcutaneous tissue were desensitized with a subcutaneous (SC) infiltration of 100 mg lidocaine (Lidocaine 2% B. Braun, Barcelona, Spain). After 5 min, a 15 cm, 18G spinal needle (MILA Spinal Needle, MILA International, Boone, KY, USA), prefilled and connected to a 60 mL syringe (Omnifix, B. Braun, Barcelona, Spain), was inserted in a craniocaudal direction at approximately 45° to the skin, with the bevel facing upwards. The needle was advanced under continuous ultrasound guidance until the tip contacted the dorsal aspect of the transverse process [25] (Figure 2).

**Figure 3:**
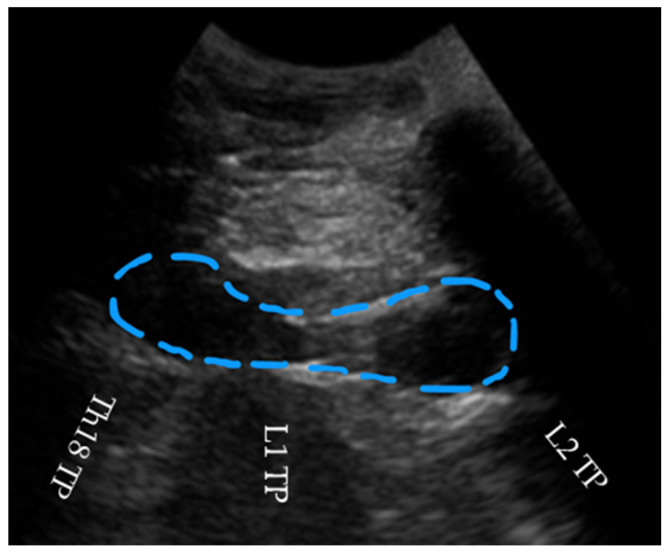
The hydrodissection (blue dashed line) with the volume injected between the erector spinae muscular complex and the eighteenth thoracic (Th18), first (L1), and second lumbar (L2) transverse processes (TPs).

After confirming negative aspiration, needle position was verified by injecting 2 mL of local anesthetic and observing hydrodissection between the longissimus muscle and the transverse process, confirming correct location under the thoracolumbar fascial plane (Figure 3; Video S1). A volume of 0.1 mL/kg containing 4 mg/kg of lidocaine (LID group) or 0.9% saline solution (Sodium Chloride 0.9%, B. Braun, Barcelona, Spain) (SS group) was then injected slowly.

All injections were performed by an experienced anesthesiologist (M.d.M.G). Technique feasibility was evaluated based on injection time (from initial probe contact to completion of injection) and ultrasound image quality. Image quality was classified as excellent (entire shaft and tip visualized), good (only tip clearly visualized), or poor (tip not visualized) [33]. The horse’s reaction during injection (any movement or behavioral response that impeded the procedure) was recorded as “Yes” or “No.”

The injection site was then marked, and the same procedure was repeated on the contralateral side. For randomization, two sets of papers were used before each injection: one set of 24 papers (12 labeled “LID” and 12 “SS”) determined the treatment group, and a second set (12 labeled “left” and 12 “right”) determined the side. Each injection was limited to a maximum of 5 min.

### 2.3. Pinprick Stimulation and Ataxia Assessment

**Figure 4:**
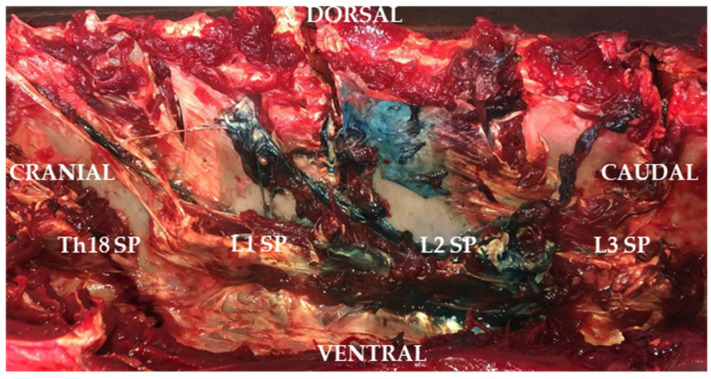
The spread pattern after administering 0.1 mL/kg of the saline solution–methylene blue 1:1 in an adult horse. SP: spinous process.

The metamers from Th17 to L5 were considered for potential involvement, based on known differences in distribution between cadaveric and in vivo models. In addition, this type of block appears to have a general tendency towards more caudal spread [27]. This decision was also supported by our unpublished personal data, suggesting possible spread extending one metamer cranial and two caudal to the injection point (Figure 4). Each metamer was identified by palpation and marked for subsequent assessment of block efficacy. The thoracolumbar block’s efficacy was evaluated by measuring the craniocaudal (CC), from Th17 to L5, and dorsoventral (DV) spread using pinprick stimulation. Four DV points were tested per metamer: 2 cm and 10 cm ventral to the dorsal midline and at the level of the iliac crest and the stifle joint.

The assessments were performed using a straight 14 cm Kelly forceps (Aesculap, Melbourne, Australia). Two investigators (F.M-B. and C.D.F.) conducted all stimulations. Briefly, the forceps was applied perpendicularly to the skin at each test point and locked to the first ratchet. A response was considered positive (sensitive) or negative (desensitized) based on the presence or absence of one or more of the following behavioral signs: panniculus muscle contraction, visible skin twitching, increased muscle tone, tail movement, kicking, or stepping away. Only one pinprick was performed per point at each time interval. Except for baseline, pinprick testing across metamers was performed in a randomized order, with the direction of testing varying between caudal-to-cranial and cranial-to-caudal and between dorsal-to-ventral and ventral-to-dorsal. Baseline testing was conducted 15–30 min prior to the first TL-ESP injection. Evaluations were repeated at 5 (T5), 10 (T10), 15 (T15), 30 (T30), 60 (T60), 90 (T90), and 120 (T120) minutes after each TL-ESP injection. If a negative response persisted at T120, evaluations were continued every 30 min until all previously negative points became positive.

The second TL-ESP injection was performed immediately after completing the first. During baseline testing, one side (left or right) of the thoracolumbar region was tested first. Stimulation started at L5 and proceeded cranially, assessing each intervertebral space until Th17 was reached. The contralateral side was then tested using the same protocol. Ataxia was evaluated subjectively by the same two investigators (F.M-B. and I.N.) at each time point using a 4-point ordinal scale (4 = severe; 3 = moderate; 2 = mild; 1 = none) [34].

### 2.4. Statistical Analysis

Statistical analyses were performed using the open-source software JASP (Version 0.19). Normality of data was assessed using the Shapiro–Wilk test. Quantitative variables with normal distribution are presented as mean ± standard deviation (SD), while non-normally distributed variables are expressed as median and interquartile range (IQR). Categorical (nominal) variables are expressed as percentages.

As part of the assessment of block feasibility, the proportion of horses showing cutaneous desensitization in each evaluated area was analyzed as the primary quantitative variable. Differences between groups were assessed using the Wilcoxon rank-sum test. Statistical significance was set at *p* < 0.05.

## 3. Results

The volume administered per injection point was 45.4 ± 7 mL.

### 3.1. Block Feasibility

All injections were well tolerated by the horses (24/24; 100%). The quality of the ultrasound visualization was rated as excellent—where the entire shaft and needle tip were visible—in 95.8% of cases (23/24) and good—only the needle tip was visualized—in 4.2% (1/24), with no significant difference between groups (*p* = 0.999). The total median injection time was 2.5 min (IQR: 2–3), with no significant differences observed between groups (*p* = 0.114).

### 3.2. Block Efficacy

**Figure 5:**
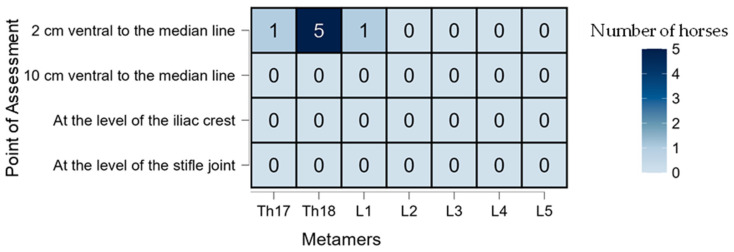
A heatmap showing the number of horses (*n* = 12) that exhibited any degree of desensitization in the saline solution (SS) group during the first 90 min. The *X*-axis represents the craniocaudal (CC) distribution expressed in metamers. The *Y*-axis represents the dorsoventral (DV) distribution expressed as 2 and 10 cm ventral to the midline and at the level of the coxal tuberosity and stifle joint.

**Figure 6:**
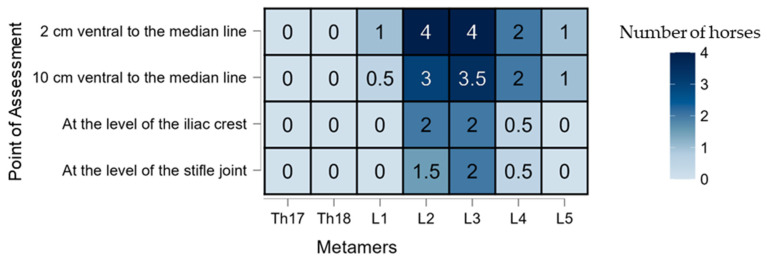
A heatmap showing the number of horses (*n* = 12) that exhibited any degree of desensitization in the lidocaine (LID) group during the first 90 min. The *X*-axis represents the craniocaudal (CC) distribution expressed in metamers. The *Y*-axis represents the dorsoventral (DV) distribution expressed as 2 and 10 cm ventral to the mean line and at the level of the iliac crest and stifle joint.

Significant differences in desensitization were found between groups, with a higher percentage of horses showing sensory loss in the LID group compared to the SS group (*p* = 0.001). All horses receiving lidocaine (12/12; 100%) exhibited desensitization at one or more assessment points, whereas only 75% (9/12) of horses in the SS group showed any desensitization. In the SS group, the effect was primarily restricted to the Th18 metamer, specifically at the 2 cm DV position ventral to the midline (Figure 5), and had a shorter duration (Table 1). In contrast, the LID group demonstrated a broader pattern of desensitization, extending both ventrally (DV) and caudally (CC) (Figure 6). For descriptive purposes, clinical relevance was considered when ≥50% of horses exhibited cutaneous desensitization at a specific time point. This threshold was used as an arbitrary yet practical indicator of potential block efficacy.

The onset of desensitization appeared significantly earlier in the SS group than in the LID group for the 2 cm DV point. However, in the LID group, the effect lasted significantly longer at the 2 and 10 cm DV points (Table 1).

**Figure 7:**
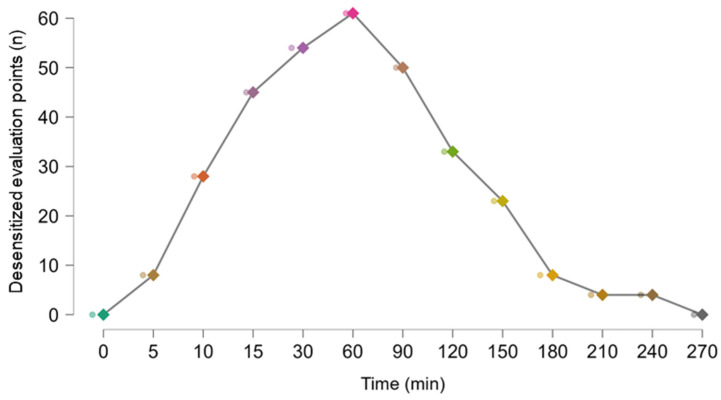
A line graph showing the number of desensitized evaluation points over time in the lidocaine (LID) group. The total possible number of desensitized evaluation points in the LID group was 336.

**Figure 8:**
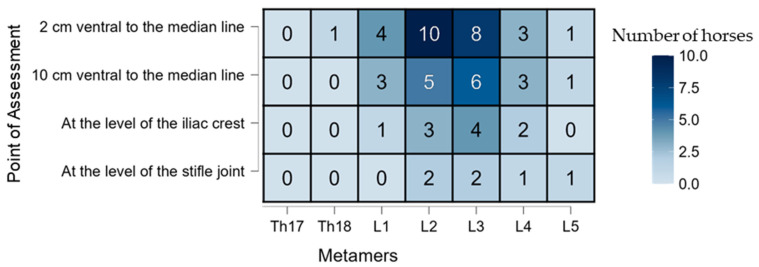
A heatmap showing the distribution pattern (%) of the desensitization in the lidocaine (LID) group (*n* = 12) at the point of maximum effect (60 min after blocking). The *X*-axis represents the craniocaudal (CC) distribution expressed in metamers. The *Y*-axis represents the dorsoventral (DV) distribution expressed as 2 and 10 cm ventral to the mean line and at the level of the iliac crest and stifle joint.

**Figure 9:**
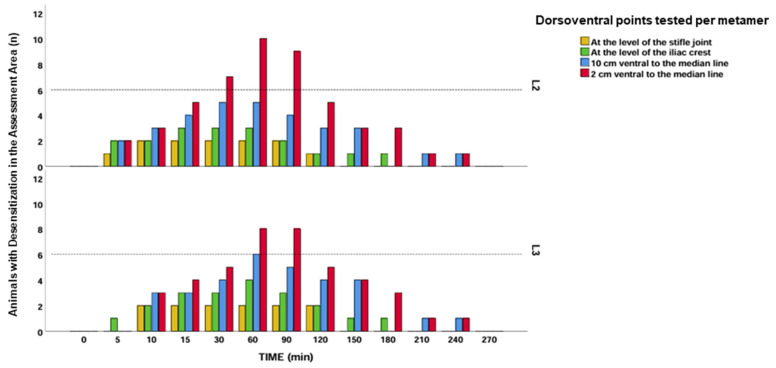
A bar graph showing the number of horses in the lidocaine group that showed a negative response at the different points tested at L2 and L3. Each bar represents each of the dorsoventral points expressed in a different color.

In the LID group, the maximum desensitization effect was observed at 60 min, with a broader ventral (DV) and craniocaudal (CC) spread (Figure 7). At this time point, 10 out of 12 horses (83.3%) showed desensitization at the L2 metamer, 2 cm from the midline (Figure 8). The block was considered clinically effective at L2 (2 cm DV) between 30 and 90 min and at L3 (2 cm DV) between 60 and 90 min. At the 10 cm DV point, this criterion was only met at L3 at 60 min (Figure 9).

## 4. Discussion

This preliminary study confirmed that the ultrasound-guided TL-ESP block is technically feasible in standing horses. The injection was performed at L1 using 0.1 mL/kg of lidocaine (4 mg/kg), with a clear needle visualization in nearly all cases and a short injection time (2–3 min). The anatomical landmark based on the transverse process width difference between Th18 and L1 was consistent and easy to identify. Desensitization was achieved dorsally in up to 100% of horses, with minimal ventral spread and no significant adverse effects.

Delgado et al. [27] reported that identifying sonographic landmarks post-mortem was feasible but hindered by emphysema. In contrast, this in vivo study achieved an excellent ultrasound image quality in 23/24 injections. Horses tolerated the procedure under the SC block, and when the needle visibility was suboptimal, punctures were repeated rather than using the “in-and-out” technique [17]. In addition, the long-axis ergonomics method [35] improved the needle visualization. The hydrodissection confirmed a correct placement in all cases, but the injectate spread was often irregular. Given previous discrepancies between the dye spread and nerve staining [27], correlating ultrasound patterns with pinprick results could be of interest. The hydrodissection was consistently observed between the longissimus dorsi and the transverse processes, confirming the correct fascial plane injection. However, the injectate distribution appeared irregular in most cases. Given prior findings by Delgado et al. [27], showing discrepancies between the dye spread and nerve staining, a correlation between the ultrasound spread and pinprick response may warrant further investigation.

Regarding efficacy, desensitization at the 2 cm DV point was consistently observed in the LID group, especially at the peak effect time (T60), as shown in Figure 8. Interestingly, the SS group also showed negative pinprick responses at this point (Figure 5), likely due to the SC infiltration interfering with the assessment. The SC injection was necessary since horses were evaluated unsedated to avoid the antinociceptive effects of α_2_-agonists [36]. The onset of desensitization appeared faster in the SS group, possibly because the SC block was already acting at early time points. A similar pattern would have been expected in the LID group, since both received the same SC infiltration; however, this was not observed, suggesting a possible masking effect by the deeper TL-ESP block or individual variability in responses. The LID group showed a longer-lasting effect, likely due to the deposition of lidocaine in a poorly vascularized fascial plane, which slows the absorption and prolongs the action [37]. Craniocaudal (CC) desensitization patterns also differed: in the SS group, the desensitization was mainly at Th18—consistent with a more cranial SC block site—while in the LID group, the caudal spread of the block led to desensitization primarily at L2 and L3, aligning with previous observations of this block’s distribution [38]. Administering 0.1 mL/kg of lidocaine in this study resulted in a mean spread of two metamers in the LID group, which is considerably less than the five metamers reported by Delgado et al. [27] using 0.3 mL/kg. The ventral spread was also limited, with a maximum success rate of 2 out of 12 horses at the iliac crest DV point at T120. These findings are consistent with those of Delgado et al. [27], who observed ventral rami staining in only three out of 24 specimens. Notably, there was considerable individual variability in the response, as also reported in previous studies. It is important to note that this study was conducted in vivo, and factors such as spontaneous ventilation may have influenced the drug distribution via pressure gradients or bulk flow mechanisms [39]. Multiple factors may contribute to the variability in the spread, including the layered orientation of the paraspinal muscles, the dense intertransverse connective tissue acting as a barrier to diffusion, and even the potential neuroimmune function of lymphatic vessels [40]. Moreover, the injection speed—which was not standardized in this study—could have affected the block’s efficacy, as previously suggested for epidural injections [41,42].

Despite these findings, some limitations must be acknowledged. Efficacy assessments were subjectively performed by a single evaluator, which may have introduced an observer bias. Additionally, an a priori sample size calculation was not conducted as this study was primarily descriptive in nature. The pinprick stimulation test, commonly used to evaluate the superficial nociceptive function via Aδ and C fibers, has notable limitations for assessing deep or visceral pain. Visceral pain tends to be diffuse, poorly localized, and often accompanied by referred pain or autonomic signs—features that differ from somatic pain and are particularly relevant in large animals like horses [43]. Nevertheless, the pinprick test proved useful in determining the extent of the desensitization achieved by the TL-ESP block, showing consistent differences between the LID and SS groups. However, the inability to assess the visceral analgesia remains a limitation. Future studies incorporating quantitative sensory testing or neurophysiological techniques could provide a more complete understanding of the block’s efficacy, especially in deeper anatomical structures [43]. Furthermore, since this test relies on behavioral responses, the individual variation in the temperament and interpretation of stimuli can introduce variability. For this reason, the horse temperament was assessed before initiating the study.

The findings of this study suggest that the TL-ESP block is effective in desensitizing areas adjacent to the spine by targeting the dorsal rami, which are implicated in conditions such as DSPs and osteoarthritis of the articular process joints [1,2]. Given the predominantly caudal distribution pattern observed, injecting cranially relative to the target metamer may improve efficacy [38]. Moreover, because the DSP typically involves multiple vertebrae, future research should explore the use of larger injection volumes and multiple injection sites to achieve broader and longer-lasting desensitization. Finally, the exact mechanism of action of the ESP block remains unclear and warrants further investigation.

## 5. Conclusions

In this study, the ultrasound-guided thoracolumbar erector spinae plane (TL-ESP) block using 0.1 mL/kg of injectate containing 4 mg/kg of lidocaine was demonstrated to be both feasible and safe to perform in standing adult horses. The block proved effective only in areas adjacent to the spine, close to the injection site. However, potential interference from the SC infiltration and the observed variability in both the craniocaudal and dorsoventral spread limit the conclusions regarding its efficacy. Further studies evaluating larger injection volumes and multiple injection sites are warranted to optimize the clinical utility of this technique.

## Figures and Tables

**Table 1:** A table showing the duration of the block (onset and end) of the saline solution (SS group) and lidocaine (LID group) groups. Data are presented as median (interquartile range, IQR) and are ordered according to dorsoventral (DV) points.

	Start of Action (Min)			End of Action (Min)		
DV Points	SS Group	LID Group	Wilcoxon Test	SS Group	LID Group	Wilcoxon Test
2 cm	5 (1.25–25)	22.5 (11.25–60)	*p* = 0.024 (20 min: 95%CI 2.5–35 min)	60 (3.75–60)	135 (120–210)	*p* = 0.003 (112.5 min: 95%CI 75–150 min)
10 cm	0 (0)	15 (5–52.5)	*p* = 0.007 (22.5 min: 95%CI 5–37.5 min)	0 (0)	105 (67.5–180)	*p* = 0.005 (120 min: 95%CI 60–165 min)
Iliac crest	0 (0)	0 (0–12.5)	*p* = 0.168	0 (0)	0 (0–120)	*p* = 0.058
Stifle joint	0 (0)	0 (0–7.5)	*p* = 0.112	0 (0)	0 (0–90)	*p* = 0.102

## Data Availability

Data is available at https://github.com/v52mebaf/TLESP-experimental-horses-DATA.git (accessed on 29 July 2025).

## Supplementary Materials

The following supporting information can be downloaded at https://www.mdpi.com/article/10.3390/ani15152264/s1: Video S1: Hydrodissection between the longissimus muscle and the transverse process.

## Conference Presentation

This article is a revised and expanded version of a paper entitled “Thoracolumbar erector spinae plane block: A preliminary experimental study in horses”, which was presented at AVA Spring Meeting, Baden (Switzerland), April 2024.

## References

[1] Walmsley J.P., Pettersson H., Winberg F., McEvoy F. (2002). Impingement of the dorsal spinous processes in two hundred and fifteen horses: Case selection, surgical technique and results. Equine Vet. J..

[2] Girodroux M., Dyson S., Murray R. (2009). Osteoarthritis of the thoracolumbar synovial intervertebral articulations: Clinical and radiographic features in 77 horses with poor performance and back pain. Equine Vet. J..

[3] Wennerstrand J., Johnston C., Roethliberger-Holm K., Erichsen C., Eksell P., Drevemo S. (2004). Kinematic evaluation of the back in the sport horse with back pain. Equine Vet. J..

[4] Spoormakers T.J.P., Stefanie V., Graat E.A.M., van-Weeren R., Brommer H. (2024). Osseous pathologic changes in the lumbar region of the equine vertebral column: A descriptive post-mortem study in three breeds. Equine Vet. J..

[5] Treß D., Lischer C., Merle R., Ehrle A. (2024). International survey of equine orthopaedic specialists reveals diverse treatment strategies for horses with overriding spinous processes. Vet. Rec..

[6] Chin K.J., El-Boghdadly K. (2021). Mechanisms of action of the erector spinae plane (ESP) block: A narrative review. Can. J. Anaesth..

[7] Chin K.J., Lirk P., Hollmann M.W., Schwarz S.K.W. (2021). Mechanisms of action of fascial plane blocks: A narrative review. Reg. Anesth. Pain Med..

[8] Chin K.J., Versyck B., Elsharkawy H., Rojas Gomez M.F., Sala-Blanch X., Reina M.A. (2021). Anatomical basis of fascial plane blocks. Reg. Anesth. Pain Med..

[9] Kim D.H., Kim S.J., Liu J., Beath J., Memtsoudis S.G. (2021). Fascial plane blocks: A narrative review of the literature. Reg. Anesth. Pain Med..

[10] Lönnqvist P.A., Karmakar M. (2019). Close-to-the-nerve vs interfascial plane blocks: Sniper rifle vs shotgun—Which will hit the target most reliably?. Acta Anaesthesiol. Scand..

[11] Forero M., Adhikary S.D., Lopez H., Lopez H., Tsui C., Chin K.J. (2016). The erector spinae plane block a novel analgesic technique in thoracic neuropathic pain. Reg. Anesth. Pain Med..

[12] Restrepo-Garces C.E., Chin K.J., Suarez P., Diaz A. (2017). Bilateral continuous erector spinae plane block contributes to effective postoperative analgesia after major open abdominal surgery: A case report. A A Case Rep..

[13] Portela D.A., Campoy L., Otero P.E., Martin-Flores M., Gleed R.D. (2017). Ultrasound-guided thoracic paravertebral injection in dogs: A cadaveric study. Vet. Anaesth. Analg..

[14] Forero M., Rajarathinam M., Adhikary S., Chin K.J. (2018). Erector spinae plane block for the management of chronic shoulder pain: A case report. Can. J. Anesth..

[15] Hernandez M., Palazzi L., Lapalma J., Forero M., Chin K.J. (2018). Erector spinae plane block for surgery of the posterior thoracic wall in a pediatric patient. Reg. Anesth. Pain Med..

[16] Bugada D., Zarcone A.G., Manini M., Lorini L.F. (2019). Continuous erector spinae block at lumbar level (L4) for prolonged postoperative analgesia after hip surgery. J. Clin. Anesth..

[17] Ferreira T.H., St James M., Schroeder C.A., Hershberger-Braker K.L., Teixeira L.B.C., Schroeder K.M. (2019). Description of an ultrasound-guided erector spinae plane block and the spread of dye in dog cadavers. Vet. Anaesth. Analg..

[18] Portela D.A., Castro D., Romano M., Gallastegui A., Garcia-Pereira F., Otero P.E. (2019). Ultrasound-guided erector spinae plane block in canine cadavers: Relevant anatomy and injectate distribution. Vet. Anaesth. Analg..

[19] Medina-Serra R., Foster A., Plested M., Sanchis S., Gil-Cano F., Viscasillas J. (2021). Lumbar erector spinae plane block: An anatomical and dye distribution evaluation of two ultrasound-guided approaches in canine cadavers. Vet. Anaesth. Analg..

[20] Portela D.A., Romano M., Zamora G.A., Garcia-Pereira F., Pablo L.S., Gatson B.J., Johnson A.N., Otero P.E. (2021). The effect of erector spinae plane block on perioperative analgesic consumption and complications in dogs undergoing hemilaminectomy surgery: A retrospective cohort study. Vet. Anaesth. Analg..

[21] Alza Salvatierra D.N., Herrera Linares M.E., Motta L., Martinez M. (2021). Ultrasound-guided erector spinae interfascial plane block for spinal surgery in three cats. JFMS Open Rep..

[22] d’Anselme O., Hartnack A., Suarez Sanchez Andrade J., Alfaro Rojas C., Ringer S.K., de Carvalho Papa P. (2022). Description of an ultrasound-guided erector spinae plane block and comparison to a blind proximal paravertebral nerve block in cows: A cadaveric study. Animals.

[23] d’Anselme O., Gamsjäger L., Hartnack A. (2024). Clinical application of a described erector spinae plane block for locoregional anaesthesia technique in a cow undergoing standing laparotomy. Vet. Rec. Case Rep..

[24] Rodriguez A., Medina-Serra R., Lynch N., Veres-Nyeki K. (2022). Erector spinae block as part of a multimodal analgesic approach in an anaesthetised horse undergoing dorsal spinous process ostectomy and desmotomy. Vet. Rec. Case. Rep..

[25] Chiavaccini L., Calvancanti M., De Gasperi D., Portela D.A. (2022). Clinical efficacy of ultrasound-guided bilateral erector spinae plane block for standing lumbar spinous osteotomy in a horse. Vet. Anaesth. Analg..

[26] Perez B.R., Hawkins A., Fiske-Jackson A., Jimenez C.P. (2023). Opioid-free anaesthesia protocol for standing spinal surgery in a horse. Vet. Rec. Case Rep..

[27] Delgado O.B.D., Louro L.F., Rocchigiani G., Verin R., Humphreys W., Senior M., Campagna I. (2021). Ultrasound-guided erector spinae plane block in horses: A cadaver study. Vet. Anaesth. Analg..

[28] Burns P.B., Rohrich R.J., Chung K.C. (2011). The levels of evidence and their role in evidence-based medicine. Plast. Reconstr. Surg..

[29] Arun N., Singh S. (2020). Is ESP block an answer for upper abdominal surgeries where epidural analgesia can’t be used?. J. Anaesthesiol. Clin. Pharmacol..

[30] Kamel A.A.F., Amin O.A.I., Ibrahem M.A.M. (2020). Bilateral Ultrasound-Guided Erector Spinae Plane Block Versus Transversus Abdominis Plane Block on Postoperative Analgesia after Total Abdominal Hysterectomy. Pain Physician.

[31] Henneke D.R., Potter G.D., Kreider J.L., Yeates B.F. (1983). Relationship between condition score, physical measurements and body fat percentage in mares. Equine Vet. J..

[32] Donaldson L.L., Dunlop G.S., Holland M.S., Burton B.A. (2000). The Recovery of Horses From Inhalant Anesthesia: A Comparison of Halothane and Isoflurane. Vet. Surg..

[33] Otero P.E., Portela D.A., Fuensalida S.E., Romano M., Cavalcanti M., Texeira J.G., Jones R., Guerrero J.A. (2022). Pericapsular hip desensitization in dogs. A cadaveric study and case series. Vet. Anaesth. Analg..

[34] Hector R., Rezende M.L., Mama K.R., Hess A.M. (2020). Recovery quality following a single post-anaesthetic dose of dexmedetomidine or romifidine in sevoflurane anaesthetised horses. Equine Vet. J..

[35] Di Franco C., Tayari H., Nardi S., Briganti A. (2021). Along or across the visual axis: A comparison of two ultrasound screen, needle, and transducer orientation techniques. Vet. Anaesth. Analg..

[36] Küls N., Trujanovic R., Otero P.E., Larenza-Menzies M.P. (2020). Ultrasound-Guided Transversus Abdominis Plane Block in Shetland Ponies: A Description of a Three-Point Injection Technique and Evaluation of Potential Analgesic Effects. J. Equine Vet. Sci..

[37] Elsharkawy H., Pawa A., Mariano E.R. (2018). Interfascial Plane Blocks: Back to Basics. Reg. Anesth. Pain Med..

[38] Otero P.E., Fuensalida S.E., Russo P.C., Verdier N., Blanco C., Portela D.A. (2020). Mechanism of action of the erector spinae plane block:dsitribution of dye in a porcine model. Reg. Anesth. Pain Med..

[39] Stecco C., Macchi V., Porzionato A., Duparc F., De Caro R. (2011). The fascia: The forgotten structure. Ital. J. Anat. Embryol..

[40] Ishizuka K., Sakai H., Tsuzuki N., Nagashima M. (2012). Topographic anatomy of the posterior ramus of thoracic spinal nerve and surrounding structures. Spine.

[41] Kanai A., Suzuki A., Hoka S. (2005). Rapid injection of epidural mepivacaine speeds the onset of nerve blockade. Can. J. Anesth..

[42] Son W.G., Jang M., Junghee Y., Lee L.Y., Lee I. (2014). The effect of epidural injection speed on epidural pressure and dsitribution of solution in anaesthetized dogs. Vet. Anaesth. Analg..

[43] Kuhlmann L., Olesen S.S., Grønlund D., Olesen A.E., Wilder-Smith O.H.G., Drewes A.M. (2019). Clinical pain phenotype associates with sensory testing results in chronic pancreatitis. Clin. J. Pain.

